# Advanced Progress of Non-Stoichiometric Transition Metal Sulfides for Sensing, Catalysis, and Energy Storage

**DOI:** 10.3390/nano15161237

**Published:** 2025-08-13

**Authors:** Xuyang Xu, Mengyang Zhang, Jincheng Wu, Ziyan Shen, Yang Liu, Longlu Wang

**Affiliations:** 1 School of Internet of Things, Nanjing University of Posts and Telecommunications (NJUPT), Nanjing 210023, China; b22100626@njupt.edu.cn; 2 Institute of Flexible Electronics (Future Technology), College of Electronic and Optical Engineering, Nanjing University of Posts and Telecommunications (NJUPT), Nanjing 210023, China; b22020220@njupt.edu.cn (M.Z.); b24020616@njupt.edu.cn (J.W.); b24020617@njupt.edu.cn (Z.S.); 1223024939@njupt.edu.cn (Y.L.)

**Keywords:** Mo_2_S_3_, W_2_S_3_, NiMo_3_S_4_, Mo_6_S_8_, Mo_6_S_6_, sensor, hydrogen evolution reaction, ion batteries

## Abstract

Beyond the extensively studied two-dimensional transition metal dichalcogenides, a wide range of non-stoichiometric transition metal sulfides, such as molybdenum sulfides and tungsten sulfides (Mo_2_S_3_, W_2_S_3_, Mo_6_S_8_, Mo_6_S_6_, NiMo_3_S_4_), have attracted significant attention for their promising applications in sensing, catalysis, and energy storage. It is necessary to review the current advanced progress of non-stoichiometric transition metal sulfides for various applications. Here, we systematically summarize the synthesis strategies of the non-stoichiometric transition metal sulfides, encompassing methods such as the molten salt synthesis method, high-metal-content growth strategy, and others. Particular emphasis is placed on how variations in the metal-to-sulfur ratio give rise to distinct crystal structures and electronic properties, and how these features influence their conductivity, stability, and performance. This review will deepen the understanding of the state of the art of non-stoichiometric transition metal sulfides, including the synthesis, characterization, modification, and various applications.

## 1. Introduction

Two-dimensional transition metal dichalcogenides (TMDs) possess tunable electronic structures, excellent catalytic properties, and strong excitonic effects, which have been widely applied in various fields [1,2,3,4,5,6,7,8]. Beyond the TMDs, a wide range of non-stoichiometric transition metal sulfides (NSTMSs) have also been applied in the field of sensing, catalysis, and energy storage due to their unique physicochemical properties. A large number of NSTMSs, such as Mo_2_S_3_, W_2_S_3_, Mo_6_S_8_, Mo_6_S_6_, NiMo_3_S_4_, have been explored [9,10,11,12,13,14,15]. Their electronic structures can be readily tailored through intrinsic defects, intercalated atoms, or elemental doping [16,17,18,19,20]. Compared to their stoichiometric counterparts, these NSTMSs also exhibit superior performance in sensing, catalysis, and energy storage applications. Despite their non-stoichiometric composition, these transition metal chalcogenides retain the layered crystal structure and weak interlayer van der Waals interactions characteristic of their stoichiometric counterparts, enabling them to be similarly processed into nanosheets through standard exfoliation or chemical methods. These unique properties have made NSTMSs a research hotspot in materials science, driving the development of novel conceptual devices [21,22,23,24,25].

Advanced progress of NSTMSs for sensing, catalysis, and energy storage has been made in recent years. Zhao et al. fabricated Mo_2_S_3_ nanowire grids via liquid–liquid interfacial self-assembly, which exhibited uniform morphology and exceptional flexibility, demonstrating outstanding performance in flexible strain sensors and high-energy-density full-cell systems [26]. Tan et al. proposed replacing Mo_6_S_8_ synthesized by traditional methods with Mo_6_S_8_ encapsulated in carbon fiber (Mo_6_S_8_@CF). This approach solves the problem of Mo_6_S_8′_s tendency to aggregate and sinter at high temperatures, which reduces catalytic efficiency, while significantly improving its performance in nitroaromatic hydrocarbon electroreduction [27]. Liu et al. constructed Mo_6_S_6_ nanostructures through electron beam lithography. Though not yet practically applied, it exhibits theoretically high catalytic potential in electrocatalytic hydrogen evolution due to its unique electronic states and surface-active site distribution [28]. The applicability of NSTMSs further extends to magnetic systems and photonic platforms [29]. Advancements in novel synthesis techniques have provided fresh insights for structural optimization and performance enhancement [30,31,32,33,34,35,36,37]. These innovative synthesis approaches not only address the critical challenge of preparing NSTMSs but also establish a material foundation for performance optimization across diverse application scenarios [38,39,40,41,42,43,44].

In this work, we systematically summarize and analyze the synthesis strategies, structural diversity, and functional applications of non-stoichiometric transition metal sulfides. First, we provide a comprehensive overview of the synthetic approaches and applications of M_2_S_3_ (M = Mo, W) and its based composites, highlighting their promising potential in sensor technologies and catalysis technologies. Second, we further explored M_6_S_8_ compounds, with a particular focus on Mo_6_S_8_ due to its high conductivity and reversible electrochemical intercalation, demonstrating significant potential for applications in lithium-ion batteries. Finally, we selected Mo_6_S_6_ as a representative of the important transition metal sulfide M_6_S_6_ family, providing a detailed discussion on its structural formation mechanisms and synthesis methods, while highlighting its promising properties. By summarizing the synthetic approaches and functional applications of these transition metal sulfides, this work contributes to a deeper understanding of their broad prospects in sensing, catalysis, and energy storage.

## 2. M_2_S_3_ and Their Applications

M_2_S_3_ (M = Mo, W) has shown broad application prospects in various fields such as sensing, catalysis, electrochemical energy storage, and electronic devices. The structural characteristics and functional applications of different forms of M_2_S_3_ and M_2_S_3_ matrix composites are systematically analyzed, with special attention to their advantages and future directions in sensor, catalyst, and energy storage applications [45]. Notably, nanowire-networked Mo_2_S_3_ exhibits superior sensitivity and selectivity in energy storage and biosensing applications due to its high conductivity and open framework, which facilitates efficient electron transport pathways. Layered Mo_2_S_3_ nanosheets with adjustable layer spacing have been widely used in humidity sensors. At the same time, existing studies have shown that W_2_S_3_ has been widely used as a high-efficiency catalyst in hydrogen evolution reactions. In order to further broaden the horizons of hydrogen evolution reaction catalysts, we note that Mo_2_S_3_ matrix composites have abundant surface-active sites and show great potential in hydrogen evolution reactions (HER) and oxygen evolution reactions (OER), making them promising candidates for advanced catalytic applications [46]. Looking forward, the synthesis of these functional materials could be revolutionized by emerging machine learning-assisted techniques, such as Bayesian optimization for reaction parameter screening, which may enable precise control over morphology and defect engineering to further enhance their catalytic and sensing performance.

### 2.1. Ultrathin Mo_2_S_3_ Nanowire Network

**Figure 1:**
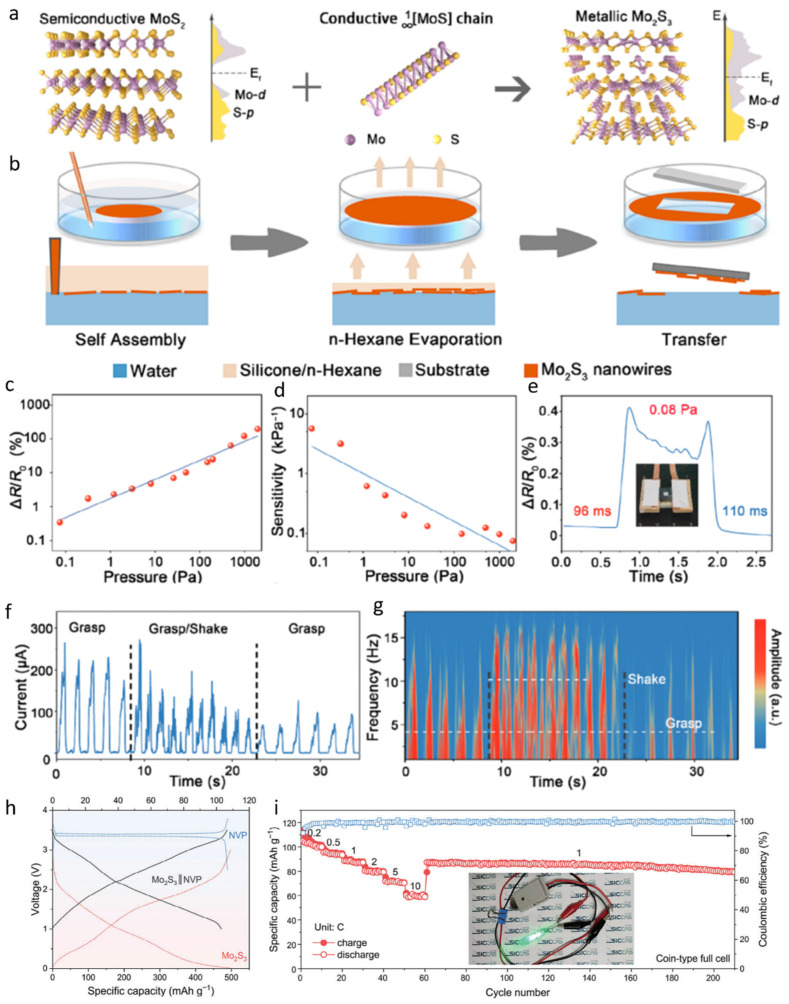
Ultrathin Mo_2_S_3_ nanowire networks designed for piezoresistive electronic skin and full-cell battery systems. (**a**) The conceptual illustration of its preparation shows the insertion of highly conductive MoS chains containing Mo−Mo zigzag structures into MoS_2_ layers. (**b**) The nanowire network is formed via liquid–liquid interface self-assembly. (**c**) The relative resistance changes in the network with increasing pressure. (**d**) The sensitivity performance of the sensor under applied pressure. (**e**) The detection limit, response, and recovery time of the sensor, along with a digital photograph from the test (the inset shows the weight of a small piece of paper with an area of 1 cm^2^). (**f**,**g**) Real-time response to current and frequency signals during Parkinson-like grasping movements. Reproduced with permission [26]. Copyright 2023, American Chemical Society. (**h**) The GCD curves of a coin-type full cell with a freestanding Mo_2_S_3_ film as the anode and NVP as the cathode. (**i**) The rate and cycling performance of the full cell, with the inset showing an LED bulb powered by the battery. Reproduced with permission [47]. Copyright 2024, Wiley-Blackwell.

At present, Mo_2_S_3_ is being widely used in the field of sensing and energy storage [47,48,49,50,51]. In order to synthesize Mo_2_S_3_, a molten salt reaction method was employed to insert conductive MoS bonds into semiconducting MoS_2_ (Figure 1a). This process led to the formation of numerous MoS clusters within the interlayers of MoS_2_. Fermi level analysis revealed that, prior to insertion, the interstitial gaps of MoS_2_ exhibited Fermi levels close to zero. However, after the incorporation of MoS bonds, a pronounced peak in the Mo-d Fermi level was observed, confirming the successful intercalation and the formation of Mo_2_S_3_. For the fabrication of large-area, ultrathin Mo_2_S_3_ nanowire networks, a liquid–liquid interfacial self-assembly technique was adopted (Figure 1b). A biphasic mixture of water and *n*-hexane was used, into which a pre-prepared Mo_2_S_3_/ethanol solution was injected at the interface. The diffusion of ethanol into the aqueous phase generated a Marangoni force, and by controlling the injection volume, the Mo_2_S_3_ nanowires were uniformly distributed along the interface. Subsequent evaporation of *n*-hexane resulted in the nanowire network floating on the water surface, from which it could be transferred onto various substrates. Additionally, the incorporation of silica gel into the *n*-hexane phase was found to enhance the stability of the assembly. The pressure-sensing performance of the Mo_2_S_3_ nanowires was systematically evaluated. As the applied pressure was increased from 0 to 1000 Pa, the relative resistance of the nanowires increased proportionally (Figure 1c), while the sensitivity decreased linearly with pressure (Figure 1d). At the lower static response limit, a relative resistance change exceeding 0.3% enabled the detection of pressure variations as small as 0.08 Pa, with a response time of 96 ms and a recovery time of 110 ms (Figure 1e). These results indicate that the nanowires can rapidly respond to and recover from subtle external pressure changes. Owing to their high adaptability and sensitivity, Mo_2_S_3_ nanowires have been applied in monitoring applications for Parkinson’s disease. The sensing mechanism is based on detecting the electrical current fluctuations caused by high-frequency oscillations superimposed during the grasping of objects. Specifically, the current profile becomes irregular during gripping and gradually returns to a smooth baseline upon cessation of oscillatory activity (Figure 1f,g). Thus, these nanowires demonstrate significant potential for real-time monitoring of motor fluctuations in Parkinson’s patients. Mo_2_S_3_ nanowires also exhibit broad applicability in the field of flexible electronics. By fabricating Mo_2_S_3_ nanofilms for use in full-cell systems, the galvanostatic charge–discharge (GCD) curves (Figure 1h) and cycling performance (Figure 1i) were analyzed. The results demonstrate that the full cell exhibits excellent electrochemical performance, enabling stable charge–discharge cycles. Moreover, even after extensive cycling, the device maintains a high capacitance retention, highlighting its promising potential for energy storage applications.

### 2.2. Layered Mo_2_S_3_ Nanosheets

While Mo_2_S_3_ nanowires have demonstrated exceptional electronic transport properties and catalytic activity owing to their distinctive one-dimensional architecture, recent research efforts have increasingly focused on two-dimensional nanonets to overcome inherent limitations in specific surface area and active site exposure. This paradigm shift is driven by the superior structural advantages of Mo_2_S_3_ nanonets—their highly porous, interconnected networks not only optimize charge transport pathways but also maximize electrolyte permeability, collectively elevating their catalytic efficiency and energy storage capabilities to new heights [52,53,54,55,56,57].

**Figure 2:**
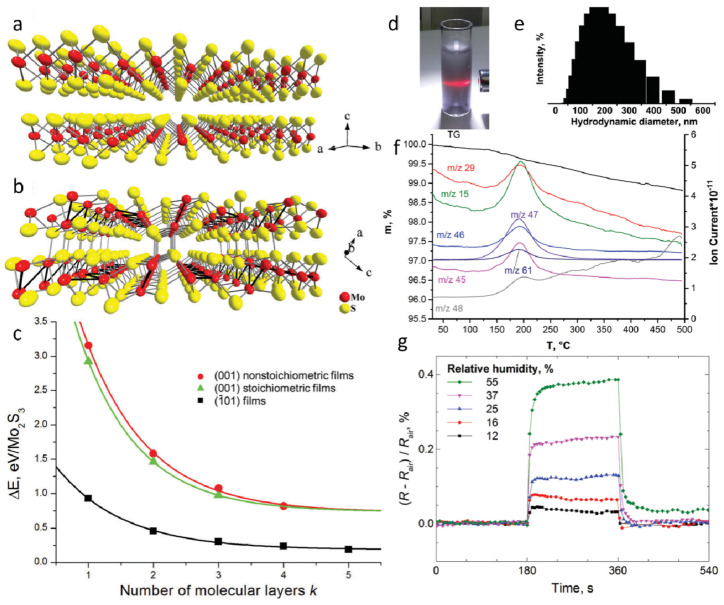
A DFT study and experimental evidence reveal the cleavage behavior of Mo_2_S_3_ under sonication in the liquid phase. (**a**) Crystal structure of layered MoS_2_. (**b**) Crystal structure of quasi-layered Mo_2_S_3_. (**c**) Relative energy variations of Mo_2_S_3_ nanosheets as a function of thickness and cleavage mode. (**d**) Photograph of Mo_2_S_3_ dispersed in an ethanol–water mixture (volume ratio 1:1). (**e**) Dynamic light scattering analysis of Mo_2_S_3_ dispersion in the ethanol–water mixture (volume ratio 1:1). (**f**) Thermogravimetric–differential scanning calorimetry (TG–DSC) and evolved gas analysis–mass spectrometry (EGA–MS) characterization of Mo_2_S_3_ precipitated from the colloidal dispersion in DMSO. (**g**) Time-dependent resistance measurements of Mo_2_S_3_ thin films in an aqueous medium. Reproduced with permission [52]. Copyright 2017, Royal Society of Chemistry.

The crystal structure of MoS_2_ consists of S–Mo–S layers arranged in a three-atomic-layer configuration, where the interlayer spacing is relatively large, and the interlayer interactions are weak (Figure 2a). In contrast, Mo_2_S_3_ features independent metallic bonds in its crystalline structure, with Mo–Mo bonding along the b-axis, rendering it significantly more stable than MoS_2_ (Figure 2b). Mo_2_S_3_ can be regarded as MoS_2_ with inserted Mo atoms, leading to a quasi-layered structure in which adjacent layers are held together by weak van der Waals interactions. Previous studies have investigated the influence of different surface terminations and thicknesses on the relative stability of Mo_2_S_3_ nanosheets (Figure 2c). Three types of Mo_2_S_3_ films were considered: (001) non-stoichiometric film, (001) stoichiometric film, and (101) film. The results indicate that Mo_2_S_3_ nanosheets with (101) surface termination exhibit the highest stability among all configurations, with their stability remaining unaffected by stoichiometry and thickness. The relative energy of all three nanosheet types increases with thickness, rapidly approaching the value of surface formation energy. The colloidal dispersions formed by ultrasonic treatment of Mo_2_S_3_ in organic media have been explored, with a series of solvents commonly used for MoS_2_ liquid-phase exfoliation tested to evaluate the suitability of Mo_2_S_3_ for similar processing. The solvent-based dispersions exhibited a grayish appearance and displayed the Tyndall effect, confirming the presence of colloidal particles in the liquid phase (Figure 2d). Dynamic light scattering (DLS) measurements of the dispersions revealed a relatively broad particle size distribution, suggesting a uniform colloidal state (Figure 2e). These findings clearly demonstrate that bulk quasi-layered Mo_2_S_3_ can be successfully exfoliated via ultrasonic treatment. To further characterize the material, thermogravimetric and mass spectrometric analyses were conducted using simultaneous thermogravimetric–differential scanning calorimetry (TG-DSC) and evolved gas analysis–mass spectrometry (EGA-MS). These analyses confirmed the presence of solvent molecules in the solid phase obtained from dimethyl sulfoxide (DMSO)-based colloidal dispersions (Figure 2f). The mass loss and corresponding ion current increase indicate that DMSO molecules are the primary gas-phase products released during this stage. The observed decomposition temperature aligns well with the boiling point of DMSO (189 °C), suggesting that Mo atoms generated upon Mo–S bond cleavage may be stabilized through complexation with solvent molecules or their residual species. Finally, Mo_2_S_3_ nanosheets were tested for their electrical resistance response to humidity variations (Figure 2g). A sensing device fabricated using Mo_2_S_3_ exhibited a significant increase in resistance upon exposure to water vapor, which rapidly returned to its initial value upon removal from the testing chamber. The humidity sensing performance of Mo_2_S_3_ nanosheets surpasses that of several other nanomaterial-based sensors, including those based on two-dimensional MoS_2_, ZnO nanowires, and graphene oxide. While these materials demonstrate certain sensitivity to humidity changes, they often suffer from slower response times or limited stability. In contrast, Mo_2_S_3_ exhibits a unique quasi-layered structure and enhanced stability, resulting in faster response and recovery times, as well as improved repeatability. Owing to its unique quasi-layered structure, high stability, and rapid humidity response, Mo_2_S_3_ represents a promising candidate for next-generation sensing materials. Future research should focus on surface functionalization to enhance solvent exfoliation efficiency, explore heterostructural integration with other two-dimensional materials, and expand its applications in flexible electronics and environmental monitoring.

Moreover, the unique quasi-layered structure and high stability exhibited by Mo_2_S_3_ nanosheets not only contribute to their outstanding humidity sensing performance but also provide a robust foundation for energy storage applications. Compared with conventional MoS_2_, which often suffers from limited electrical conductivity and relatively sluggish ion diffusion in its semiconducting 2H phase, Mo_2_S_3_ offers a higher density of active sites and more efficient electron transport pathways owing to its non-stoichiometric composition and defect-rich structure [58]. In energy storage devices, critical factors such as electrical conductivity, interfacial reaction activity, and structural stability strongly influence electrochemical performance. Mo_2_S_3_ nanosheets demonstrate significant promise for high-performance applications in lithium-ion batteries, supercapacitors, and related systems, enabling both efficient charge storage and rapid charge–discharge behavior. Furthermore, the rapid response and recovery observed in humidity sensing reflect the material’s excellent structural reversibility and chemical stability in electrolyte environments, implying favorable durability and cycling stability under electrochemical conditions. These characteristics highlight the advantages of Mo_2_S_3_ over traditional disulfide-based materials and underscore the potential of integrating sensing and energy storage functionalities in next-generation Mo_2_S_3_-based nanodevices.

### 2.3. W_2_S_3_ Nanosheets

W_2_S_3_ has emerged as a promising candidate owing to its unique crystal structure, rich defect chemistry, and favorable electrical conductivity. These features suggest strong potential for W_2_S_3_ in catalytic and energy storage applications. The following section highlights recent developments in the synthesis and functional exploration of W_2_S_3_, aiming to provide insights into its advantages over conventional layered dichalcogenides.

WS_2_ is a common catalyst with decent performance in HER, but its catalytic activity and conductivity are limited. In contrast, W_2_S_3_ features a unique non-centrosymmetric structure that induces spontaneous polarization and metallic conductivity, significantly enhancing its catalytic performance. Experimental results show that W_2_S_3_ nanosheets exhibit lower overpotentials and higher catalytic activity than WS_2_ in both acidic and alkaline media, even outperforming commercial Pt/C catalysts under alkaline conditions. Additionally, W_2_S_3_ demonstrates excellent stability in practical electrolyzers, maintaining performance during long-term high-current operation. Theoretical calculations further confirm that W_2_S_3_ has an optimal hydrogen adsorption free energy, supporting its efficient catalytic mechanism.

**Figure 3:**
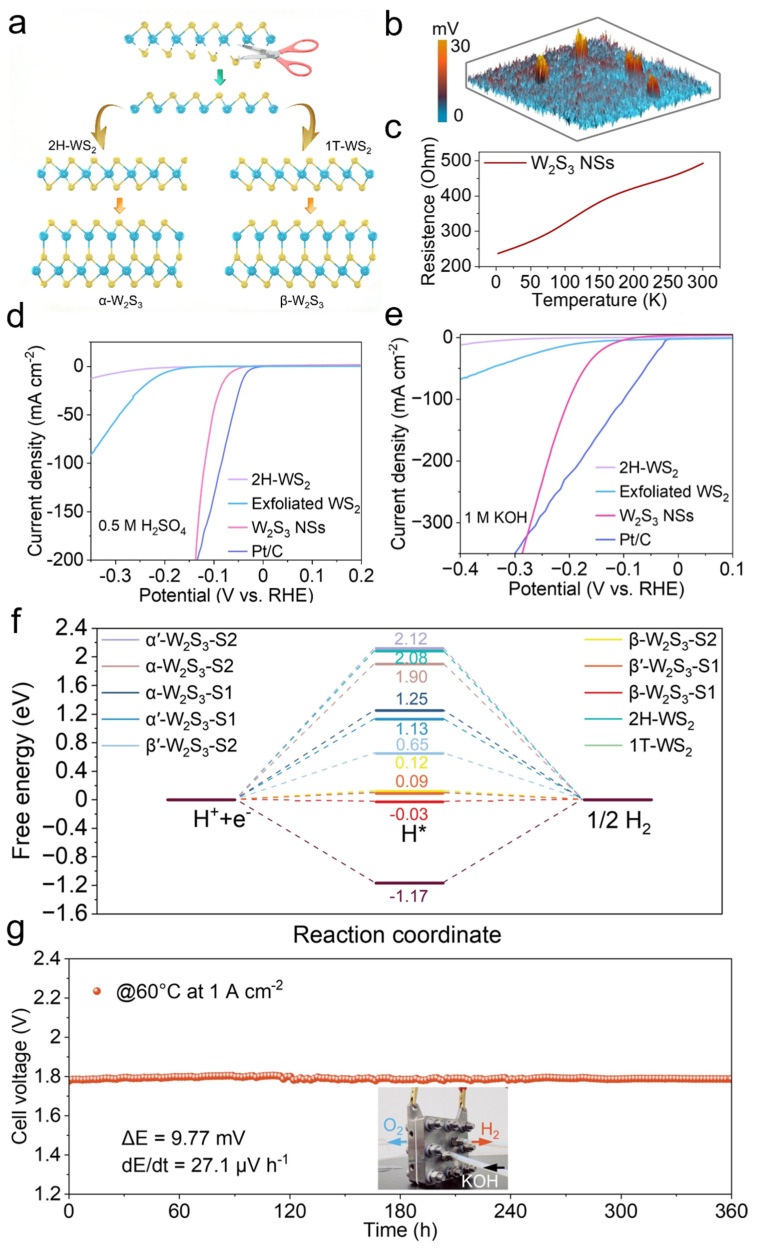
W_2_S_3_ for efficient biphasic pH hydrogen evolution reaction. (**a**) Schematic illustration of the synthesis of W_2_S_3_. (**b**) Surface potential of W_2_S_3_ nanosheets measured by Kelvin probe force microscopy (KPFM). (**c**) Temperature-dependent resistance curve of W_2_S_3_ nanosheets. (**d**,**e**) HER performance in 0.5 M H_2_SO_4_ and 1 M KOH. (**f**) Gibbs free energy diagrams of W_2_S_3_ with different structures. (**g**) Durability test of the catalyst fabricated from W_2_S_3_ nanosheets in an anion exchange membrane (AEM) electrolyzer. (Inset: Photograph of the AEM electrolyzer setup). Reproduced with permission [59]. Copyright 2024, John Wiley and Sons Ltd.

W_2_S_3_ has been extensively utilized as a catalyst in HER, significantly enhancing reaction efficiency. To synthesize W_2_S_3_, researchers initially obtained metastable 1T-phase WS_2_ (octahedral coordination) and stable 2H-phase WS_2_ (trigonal prismatic coordination) through shear exfoliation and rearrangement of WS_2_ layers [59,60,61,62,63,64]. Subsequently, monolayer WS fragments were covalently bonded with 2H- and 1T-WS_2_ layers to form α’-W_2_S_3_ and β’-W_2_S_3_ derivatives (Figure 3a). To fabricate W_2_S_3_ nanosheets (NSs), a high-metal-content growth strategy was employed, wherein the chemical ratio of tungsten to sulfur was precisely controlled to facilitate the crystallization of W_2_S_3_. The obtained W_2_S_3_ NSs were characterized by atomic force microscopy (AFM) and Kelvin probe force microscopy (KPFM) to evaluate surface potential variations. The potential mapping revealed highly distinct spherical domains with significant potential differences (Figure 3b). This surface potential disparity primarily arises from spontaneous polarization induced by the novel non-centrosymmetric structure of W_2_S_3_. Furthermore, temperature-dependent electrical measurements demonstrated that the resistance of W_2_S_3_ increased with rising temperature, indicating its metallic nature (Figure 3c). To evaluate the HER performance of W_2_S_3_ NSs in both acidic and alkaline media, a three-electrode system was utilized in 0.5 M H_2_SO_4_ and 1 M KOH solutions. The catalytic activity of W_2_S_3_ NSs was benchmarked against exfoliated WS_2_, 2H-WS_2_, and commercial Pt/C (20 wt%). In acidic solutions, W_2_S_3_ NSs exhibited significantly lower overpotentials and superior catalytic activity compared to WS_2_ and 2H-WS_2_, approaching the performance of commercial Pt/C (20 wt%) (Figure 3d). Remarkably, in alkaline solutions, W_2_S_3_ NSs outperformed commercial Pt/C (20 wt%) under high current densities (Figure 3e). To further elucidate the origin of the electrocatalytic activity of W_2_S_3_ NSs and investigate the HER catalytic mechanism, density functional theory (DFT) calculations were conducted. The Gibbs free energy of hydrogen adsorption (ΔGH*) serves as a crucial parameter for evaluating HER catalytic activity, where a ΔGH* value closer to zero indicates optimal adsorption–desorption equilibrium and enhanced HER performance. Among all examined sites, β-W_2_S_3_ exhibited the lowest ΔGH* values, suggesting its superior HER catalytic activity (Figure 3f). To assess the feasibility of large-scale HER applications, W_2_S_3_ NSs were employed as the cathode in a flow-type anion exchange membrane (AEM) electrolyzer, with commercial IrO_2_ as the anode. Under 30% KOH at 60 °C, the W_2_S_3_-based electrolyzer demonstrated excellent operational stability, maintaining a steady voltage at a current density of 1.0 A cm^−2^ for 360 h without significant degradation (Figure 3g). By leveraging a stoichiometrically controlled growth strategy coupled with theoretical structural predictions, W_2_S_3_ has been successfully developed as a highly efficient HER catalyst. Its outstanding performance in both acidic and alkaline conditions highlights its potential as a promising cathode material for large-scale hydrogen production.

### 2.4. Mo_2_S_3_ Based Composites

Through the research of the existing M_2_S_3_ in the field of catalysis, people have gradually turned their attention to the matrix composites composed of M_2_S_3_. Building upon the intrinsic properties of Mo_2_S_3_ nanostructures, recent studies have focused on the design and synthesis of Mo_2_S_3_-based composite materials to further enhance functional performance [65,66]. Among these, NiMo_3_S_4_ composites have attracted significant attention due to their synergistic effects, combining the advantageous characteristics of both Mo_2_S_3_ and nickel sulfides. This composite not only improves electrical conductivity and structural stability but also introduces additional active sites, thereby boosting catalytic and electrochemical activities.

**Figure 4:**
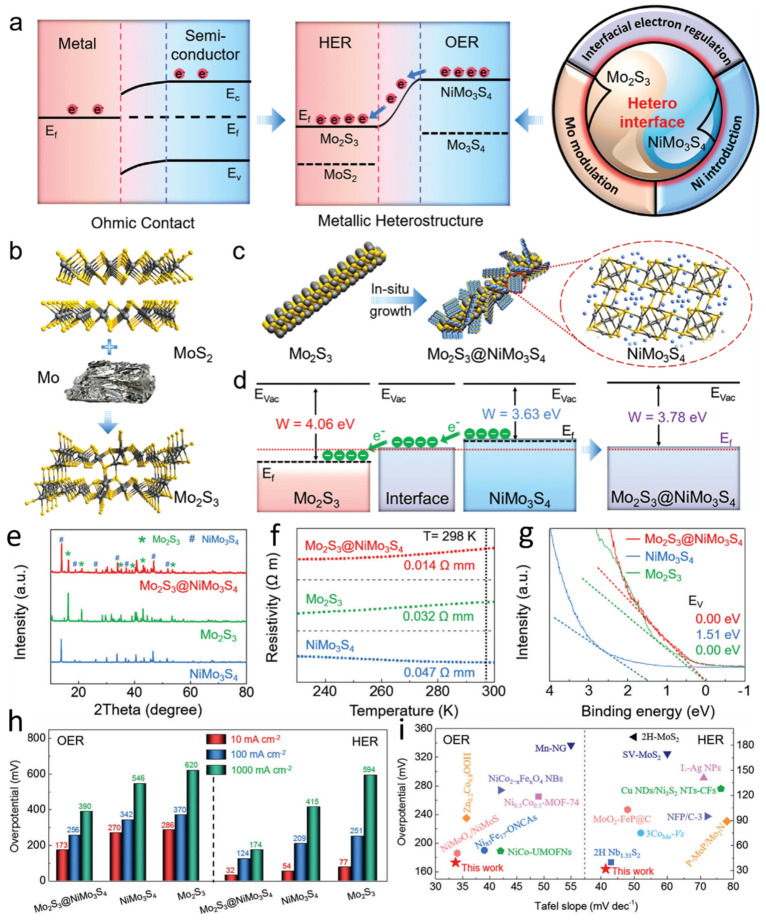
Bimetallic regulation enables high-current-density, efficient overall water splitting (**a**) Evolution of water-splitting catalysts. (**b**) Schematic illustration of the transformation from MoS_2_ to metallic Mo_2_S_3_. (**c**) Schematic representation of the epitaxial construction of Mo_2_S_3_@NiMo_3_S_4_. (**d**) Schematic diagram of the energy band structures of Mo_2_S_3_ and NiMo_3_S_4_ (Evac = vacuum energy level, Ef = Fermi level, W = work function). (**e**) Temperature-dependent resistivity curves of Mo_2_S_3_, NiMo_3_S_4_, and Mo_2_S_3_@NiMo_3_S_4_. (**f**) Ultraviolet photoelectron spectroscopy (UPS) spectra in the low-energy region for Mo_2_S_3_, NiMo_3_S_4_, and Mo_2_S_3_@NiMo_3_S_4_. (**g**) UPS photoelectron spectra of Mo_2_S_3_, NiMo_3_S_4_, and Mo_2_S_3_@NiMo_3_S_4_. (**h**) Overpotentials of Mo_2_S_3_, NiMo_3_S_4_, and Mo_2_S_3_@NiMo_3_S_4_ for the OER and HER. (**i**) Kinetics and catalytic activity of OER and HER. Reproduced with permission [65]. Copyright 2023, American Chemical Society.

Electrochemical water splitting, encompassing the hydrogen evolution reaction and oxygen evolution reaction, represents a sustainable approach for continuous hydrogen production [67,68,69,70,71,72]. However, traditional serial fabrication methods face challenges in achieving durable and rapid semiconductor heterostructures for electrode preparation. To address this issue, Mo-modulated molybdenum disulfide (MoS_2_) phases can be introduced to construct metal sulfide supports, facilitating the epitaxial growth of metal heterostructures (Figure 4a). This strategy enables the formation of Mo_2_S_3_@NiMo_3_S_4_ heterostructures, where each component plays a complementary catalytic role. The Mo_2_S_3_ phase primarily enhances HER, whereas the NiMo_3_S_4_ phase mainly promotes OER. The incorporation of Ni, which possesses asymmetric 3d orbitals, can reconfigure with the vacant 4d orbitals of Mo in the sulfide framework, driving electron transfer from the OER site to the HER site and providing abundant active sites, thereby accelerating hydrogen generation. Mo_2_S_3_ is synthesized via the reaction of MoS_2_ with Mo, while Mo_2_S_3_@NiMo_3_S_4_ is fabricated through the in situ growth of NiMo_3_S_4_ nanosheets on Mo_2_S_3_ nanorods. To synthesize Mo_2_S_3_@NiMo_3_S_4_ nanorods, an excess of Mo was first introduced to transform the semiconductor MoS_2_ into metallic Mo_2_S_3_ (Figure 4b). Subsequently, NiMo_3_S_4_ nanosheets were grown in situ on the Mo_2_S_3_ nanorod surface via a hydrothermal process, forming the Mo_2_S_3_@NiMo_3_S_4_ metal heterostructure (Figure 4c). The high Fermi-level metallic Mo_2_S_3_ serves as an efficient charge transport medium at the interface. Moreover, compared to pure metallic Mo, Mo_2_S_3_ features shorter Mo–Mo bonds, which generate delocalized electrons and electronic states, thereby facilitating HER. The electronic band structures of Mo_2_S_3_, NiMo_3_S_4_, and Mo_2_S_3_@NiMo_3_S_4_ demonstrate that the formation of the heterointerface promotes favorable electron migration from NiMo_3_S_4_ to Mo_2_S_3_ (Figure 4d). The crystal structures of the synthesized samples were further characterized using X-ray diffraction (XRD) analysis. The characteristic peaks of the Mo_2_S_3_ phase confirm its successful formation, while the distinctive peaks of the NiMo_3_S_4_ phase validate the presence of NiMo_3_S_4_ (Figure 4e). These observations unequivocally confirm the successful synthesis of Mo_2_S_3_@NiMo_3_S_4_. Furthermore, the electrical conductivity of the samples was investigated via temperature-dependent resistivity measurements (Figure 4f). At 298 K, Mo_2_S_3_@NiMo_3_S_4_ exhibits significantly lower resistivity than Mo_2_S_3_ and NiMo_3_S_4_, indicating superior electrical conductivity. Additionally, the resistivity of both Mo_2_S_3_ and Mo_2_S_3_@NiMo_3_S_4_ decreases with decreasing temperature, confirming their metallic nature. Ultraviolet photoelectron spectroscopy (UPS) analysis was conducted to further validate the low-energy electronic features of Mo_2_S_3_, NiMo_3_S_4_, and Mo_2_S_3_@NiMo_3_S_4_, substantiating the metallic characteristics of Mo_2_S_3_ and Mo_2_S_3_@NiMo_3_S_4_ (Figure 4g). The catalytic performance of Mo_2_S_3_, NiMo_3_S_4_, and Mo_2_S_3_@NiMo_3_S_4_ was subsequently evaluated. The overpotentials for OER and HER at 10, 100, and 1000 mA·cm^−2^ were measured in a 1.0 M KOH solution saturated with N_2_ at room temperature, using Mo_2_S_3_, NiMo_3_S_4_, and commercial Pt/C as reference samples (Figure 4h). The synthesized Mo_2_S_3_@NiMo_3_S_4_ catalyst exhibits lower overpotentials for both OER and HER, outperforming commercial RuO_2_, Pt/C, and recently reported electrocatalysts (Figure 4i).

Mo_2_S_3_@NiMo_3_S_4_ demonstrates exceptional OER and HER electrocatalytic performance in alkaline media, primarily due to the high activity and electrical conductivity provided by interfacial electronic engineering within its metal heterostructure [73]. This study addresses the limitations of traditional catalysts in terms of conductivity and activity through interface electronic modulation and metallic heterostructure design, offering a novel strategy for developing highly efficient and stable bifunctional electrocatalysts.

## 3. M_6_S_8_ and Their Applications

While M_2_S_3_ and its composite materials have demonstrated promising properties in sensing, catalysis, and energy storage, M_6_S_8_ has also attracted widespread attention. In this context, Mo_6_S_8_, a representative member of the Chevrel-phase family, has emerged as a particularly intriguing candidate [74,75,76,77,78]. Its unique cluster-based structure enables efficient ion intercalation, excellent structural stability, and multi-electron redox activity, making it highly attractive for applications in advanced batteries and electrocatalysis. Recent advances in AI-driven characterization (e.g., convolutional neural network-based defect analysis) are expected to provide deeper insights into the structure-activity relationships of these Chevrel-phase materials, particularly in understanding their exceptional ion transport mechanisms at atomic scales. The following section discusses recent advances in the use of M_6_S_8_ for energy-related applications, highlighting its performance advantages and underlying mechanisms.

**Figure 5:**
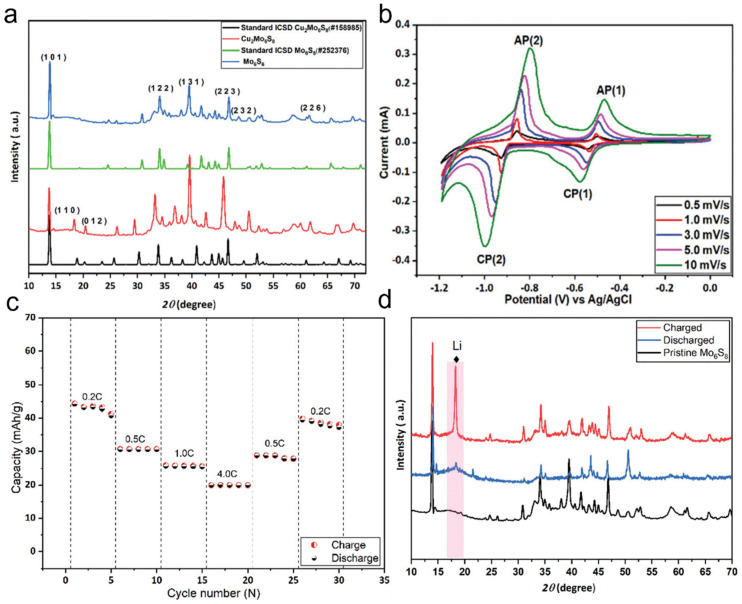
Chevrel-phase Mo_6_S_8_ as an active electrode material for lithium-ion batteries (**a**) Powder X-ray diffraction (P-XRD) patterns of Cu_2_Mo_6_S_8_ and Mo_6_S_8_ along with their corresponding standard patterns. (**b**) Cyclic voltammetry (CV) curves of the Mo_6_S_8_ electrode at different scan rates. (**c**) Rate performance of the lithium-ion battery at various current densities. (**d**) P-XRD characterization of the Mo_6_S_8_ electrode. Reproduced with permission [75]. Copyright 2022, Royal Society of Chemistry.

Chevrel-phase Mo_6_S_8_ has been widely studied as an anode material due to its unique crystal structure. The traditional preparation method involves first synthesizing Cu_2_Mo_6_S_8_ and then removing Cu to obtain Mo_6_S_8_. To obtain Cu_2_Mo_6_S_8_, CuS, MoS_2_, and elemental Mo were mixed in a stoichiometric ratio and heated at 1000 °C for 24 h, forming Cu_2_Mo_6_S_8_. The X-ray diffraction (XRD) patterns of Cu_2_Mo_6_S_8_ and Mo_6_S_8_ (Figure 5a) show that the diffraction peaks of Cu_2_Mo_6_S_8_ are highly consistent with the standard Cu_2_Mo_6_S_8_ pattern (ICSD#158985). Furthermore, the strong peak at 13.8° and the minimal peak intensity at 14.4°, corresponding to MoS_2_, indicate the high purity of the synthesized Cu_2_Mo_6_S_8_. The obtained Cu_2_Mo_6_S_8_ was then leached in 6 M HCl, yielding Chevrel-phase Mo_6_S_8_. When used as an anode material for lithium-ion batteries, Mo_6_S_8_ exhibits excellent electrochemical performance during the lithiation/delithiation process. Its cyclic voltammetry (CV) curves (Figure 5b) show a small peak separation between the anodic and cathodic reactions, suggesting fast lithium-ion insertion kinetics. The overpotential is significantly lower than that of conventional anode materials, such as VO_2_(B), V_2_O_5_, and LiTi_2_(PO_4_)_3_. This outstanding performance is primarily attributed to the nanoscale morphology of Mo_6_S_8_, which shortens the lithium-ion diffusion path and enhances kinetic performance. The rate capability and cycling stability of Mo_6_S_8_ were further evaluated by assembling Mo_6_S_8_-based coin cells with LiMn_2_O_4_ as the cathode and conducting charge–discharge tests at different rates (Figure 5c). The results indicate that the specific capacity decreases at higher charge–discharge rates, mainly due to the limited lithium-ion diffusion rate rather than electrode degradation. This finding suggests that Mo_6_S_8_ exhibits excellent structural stability. In the rate recovery test, when the charge–discharge rate was restored to its initial value, the battery capacity nearly returned to its first-cycle level, demonstrating outstanding high-rate stability. To gain deeper insight into the structural evolution of Mo_6_S_8_ during lithium-ion insertion/extraction, in situ XRD characterization was performed on cycled electrodes (Figure 5d). The XRD results reveal that during the charging process, Mo_6_S_8_ undergoes a phase transformation, forming LixMo_6_S_8_ (0 < x < 4). However, in the fully discharged state, the reappearance of a distinct diffraction peak at 18° indicates that the original structure of Mo_6_S_8_ is restored, confirming the reversibility of this phase transition. In summary, Chevrel-phase Mo_6_S_8_ exhibits exceptional lithium-ion insertion kinetics, superior rate performance, and high structural stability as an anode material for lithium-ion batteries. Its unique nanoscale morphology shortens the lithium-ion diffusion path, enhances kinetic response, and maintains excellent cycling stability even at high charge–discharge rates. Additionally, the reversible phase transition further ensures structural integrity during long-term cycling, making Mo_6_S_8_ a highly promising high-performance anode material.

**Figure 6:**
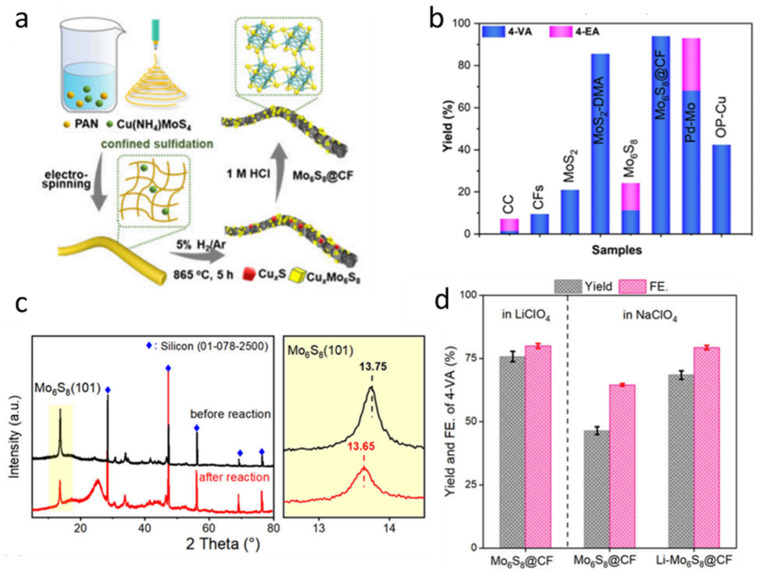
Preparation and reaction performance of in situ Li^+^-intercalated nanosized Chevrel-phase Mo_6_S_8_ catalyst. (**a**) Schematic illustration of the preparation of Mo_6_S_8_@CF (**b**) comparison of product yields using different catalysts (**c**) XRD patterns of Mo_6_S_8_ before and after the electrochemical reaction (**d**) Electrocatalytic performance of Mo_6_S_8_@CF in 0.1 M LiClO_4_ and NaClO_4_ electrolytes after 3 h, and Li-Mo_6_S_8_@CF tested in 0.1 M NaClO_4_ with 10 mM 4-NS. Reproduced with permission [27]. Copyright 2025, American Chemical Society.

However, conventional synthesis methods often lead to severe aggregation and sintering of the active materials at high temperatures, which significantly reduces the catalytic efficiency [79,80]. Therefore, Mo_6_S_8_ nanoparticles wrapped in carbon fiber (Mo_6_S_8_@CF) were selected as an alternative to overcome these limitations. Mo_6_S_8_@CF was synthesized through a combination of electrospinning and high-temperature sulfurization. Initially, a homogeneous electrospinning precursor solution was prepared by dissolving polyacrylonitrile (PAN) and copper ammonium thiomolybdate (Cu (NH_4_) MoS_4_) (Figure 6a). The solution was then subjected to electrospinning under a high-voltage electric field to form nanofibers, effectively confining the Cu (NH_4_) MoS_4_ within the PAN matrix. Subsequently, the obtained composite fibers were annealed in a 5% H_2_/Ar atmosphere at 865 °C for 5 h, during which CuxS and CuxMo_6_S_8_ intermediates were formed. Finally, these intermediates were treated with 1.0 M hydrochloric acid (HCl) to selectively remove the copper-containing phases, yielding phase-pure Mo_6_S_8_@CF. To evaluate the catalytic performance of Mo_6_S_8_@CF for the electroreduction of nitroarenes, the production efficiencies of 4-vinylaniline (4-VA) and 4-ethylaniline (4-EA) at −0.45 V were compared under identical conditions (Figure 6b). Mo_6_S_8_@CF exhibits significantly higher catalytic efficiency than other tested catalysts. The Mo_6_S_8_ structure contains cavities within its nanofibers that are capable of hosting metal ions. To verify the possible intercalation of metal ions, X-ray diffraction (XRD) analysis was conducted on the samples after the electrochemical reduction process (Figure 6c). A distinct shift in the (101) diffraction peak of Mo_6_S_8_ was observed, indicating that Li^+^ ions were successfully inserted into the Mo_6_S_8_ lattice. This intercalation leads to a noticeable lattice expansion, as evidenced by the peak shift. The electrocatalytic reduction performance of Mo_6_S_8_@CF electrodes was compared in LiClO_4_ and NaClO_4_ electrolytes, as illustrated in Figure 6d. It is evident from the figure that the reaction rate in LiClO_4_ solution is significantly higher than that in NaClO_4_. Moreover, in NaClO_4_ solution, the catalytic rate of Li-Mo_6_S_8_@CF is also markedly superior to that of Mo_6_S_8_@CF. These results fully verify the possibility of using Mo_6_S_8_@CF instead of Mo_6_S_8_ as a high-performance lithium-ion battery cathode.

**Figure 7:**
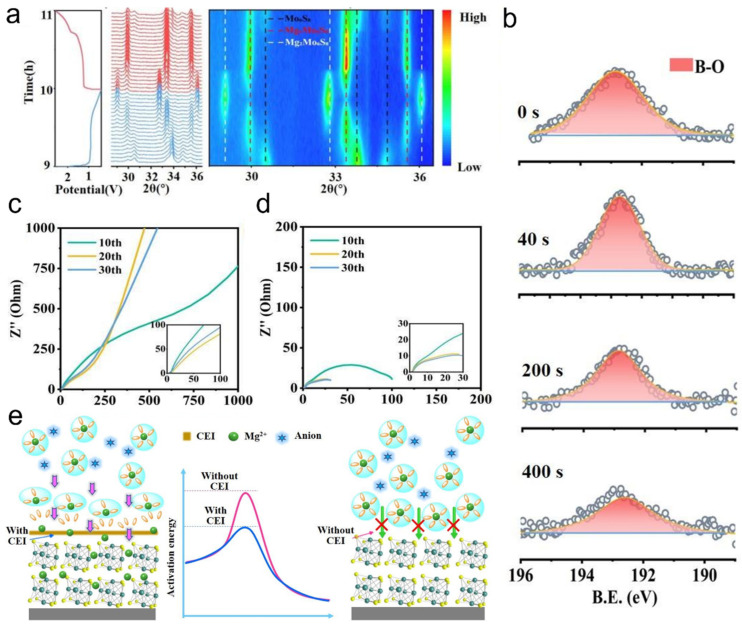
Cathode electrolyte interface (CEI) facilitates fast Mg^2+^ transport in Mo_6_S_8_: (**a**) In situ XRD patterns of the Mo_6_S_8_//Mg battery at a cutoff voltage of 2.6 V during charging. (**b**) Depth-profiled B 1s XPS spectra of the Mo_6_S_8_ cathode after cycling at 2.6 V. (**c**,**d**) EIS of Mo_6_S_8_//Mg battery at 2.0 V and 2.6 V under different cutoff voltages. (**e**) Schematic illustration of the CEI-mediated interfacial mechanism on the Mo_6_S_8_ particle surface. Reproduced with permission [81]. Copyright 2023, John Wiley and Sons Ltd.

With the strong demand for increased energy density in batteries, Mg is widely used as a metal anode due to its high volumetric specific capacity, low cost, high natural abundance, and sustainability [81,82]. The use of a chlorine-containing adsorption layer on the surface of Mo_6_S_8_ on the cathode can reduce its activation energy, thereby effectively transferring Mg^2+^. However, chloride tends to corrode the metal on the anode surface, so the use of LiBxOy as the CEI type improves the stability of the cathode. The XRD pattern of the Mo_6_S_8_//Mg cell was observed by performing a charge and discharge reaction (Figure 7a). When discharged, MgMo_6_S_8_ is formed as an intermediate product, and finally Mg_2_Mo_6_S_8_ is generated. However, when charging, Mg_2_Mo_6_S_8_ is converted into MgMo_6_S_8_, but the Mg^2+^ in it cannot continue to obtain electrons to generate Mg, because at low Mg content, Mg^2+^ has a unique ring with low potential energy, so the diffusion in MgMo_6_S_8_ is slower, so there will be coexistence of MgMo_6_S_8_ and Mo_6_S_8_ at the cathode. X-ray photoelectron spectroscopy (XPS) was performed on the Mo_6_S_8_ surface at 2.0 eV (Figure 7b). A strong B-O peak was found at around 192.5 eV, corresponding to the MgBxOy species. At the same time, it was found that the peak of the signal continued to decrease over time. This phenomenon illustrates the formation of a homogeneous CEI on the surface of the cathode made of Mo_6_S_8_. The electrochemical resistance of Mo_6_S_8_//Mg cells after different cycles was tested at voltages of 2.0 V (Figure 7c) and 2.6 V (Figure 7d), respectively. It is found that the interfacial impedance is significantly lower than that of 2.0 V at 2.6 V. And as the number of cycles increases, the interface impedance gradually decreases. Therefore, at higher voltages, Mg^2+^ is easier to transfer. Based on the above experimental process, the surface CEI working mechanism of Mo_6_S_8_ was predicted. [B(hfip)4]^−^ decompose first to form CEI and Mg^2+^ at high voltage, CEI reduces its activation energy and accelerates the movement of Mg^2+^, while Mg^2+^ on the surface of Mo_6_S_8_ without CEI is difficult to pass. Therefore, the storage performance of Mg^2+^ in the cathode can be improved (Figure 7e). Under high voltage, [B(hfip)4]^−^ decomposes to form the CEI layer, which reduces the activation energy of Mg^2+^, accelerates its migration, and improves the magnesium storage performance of Mo_6_S_8_. This study demonstrates that interfacial chemistry optimization significantly enhances the magnesium storage performance of the Mo_6_S_8_ cathode, advancing the practical development of magnesium batteries.

## 4. Other Non-Stoichiometric Metal Sulfides

Nanowires are typical 1D systems that hold potential for applications spanning from flexible nanodevices to ultrasmall functional interconnects. Among them, nanowires composed of transition metals and chalcogens, especially M_6×6_ nanowires (M = transition metal; X = chalcogen), have potential applications in 1D electron channels, spintronics, optoelectronics, and catalysis due to their authentic sub-nanometer width (<1 nm) and intrinsic metallicity [83]. However, there are still many challenges in the fabrication of M_6×6_ nanowires, especially single nanowires. Traditional methods, such as physical vapor deposition, can make the purification of individual nanowires difficult. At present, the latest research shows that the treatment of trimethyl molybdenum disulfide (TMDC) monolayer by electron beam irradiation technology is a clean and efficient preparation method [84,85,86].

**Figure 8:**
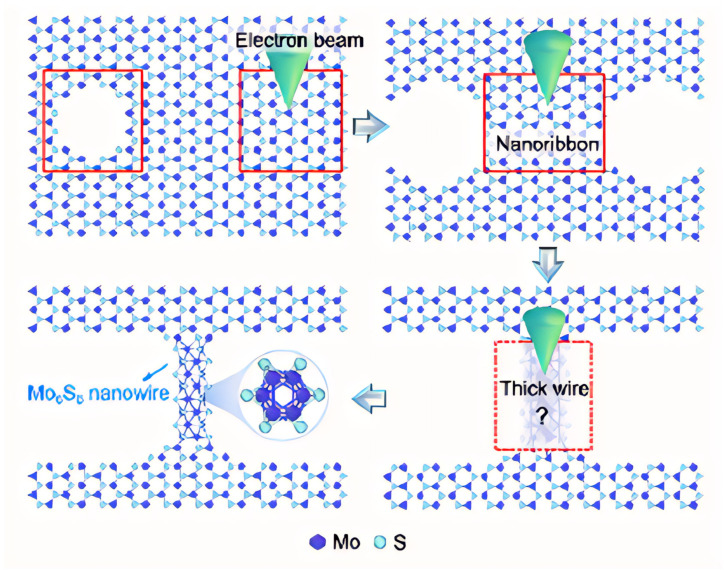
The schematic diagram of a single Mo_6_S_6_ nanowire prepared from a monolayer 1H-phase MoS_2_ material via electron beam lithography. The red box indicates the region exposed to the electron beam. Reproduced with permission [28]. Copyright 2024, American Chemical Society.

Firstly, MoS_2_ is a layered crystal, in which Mo atoms are sandwiched between two layers of S atoms, and when irradiated by a high-energy electron beam, they will preferentially excite and sputter S atoms with lower binding energy, resulting in the formation of S vacancies and defects in local areas. This initial etching disrupts the original lattice equilibrium. Then, due to the bond breakage and the uneven distribution of local energy in the defect region, the remaining Mo atoms and some S atoms began to diffuse and migrate, undergoing atomic rearrangement in the defect edge region. This atomic migration and self-organization effect prompts the atoms to be reconstructed in a new arrangement, transforming from a two-dimensional structure to a narrow nanoribbon. With the continuous action of the electron beam and the contribution of the local heat shock effect, the atoms inside the nanoribbon are further adjusted, and finally form a one-dimensional Mo_6_S_6_ nanowire with a new crystal structure, which is not only stable, but may also aggregate and fuse in the local region and evolve into a thicker wire-like structure (Figure 8).

This study reveals the unexplored mechanism of M_6×6_ single nanowire formation, which provides an important basis for further understanding the narrowing process of TMDC nanoribbons under electron irradiation [87]. Though not yet practically applied, it exhibits theoretically high catalytic potential in electrocatalytic hydrogen evolution due to its unique electronic states and surface-active site distribution.

## 5. Conclusions and Prospects

Scalable synthesis methods for non-stoichiometric transition metal sulfides have been explored to meet industrial demands in sensing, catalysis, and energy storage. These methods provide viable routes for translating laboratory-scale materials into industrial applications. Despite significant advances in their application for sensing, catalysis, and energy storage, several challenges and opportunities remain to be addressed to fully realize their potential. First, the precise correlation between crystal structure, defect engineering, and catalytic activity warrants deeper exploration. Tailoring the active sites via controlled sulfur vacancies, phase engineering, or heteroatom doping could further enhance HER performance while improving material stability under operational conditions. Studies by Denis Gentili et al. have demonstrated that defect engineering in transition metal dichalcogenides can achieve precise spatial control through advanced techniques such as electrochemical nanopatterning [88]. Second, these non-stoichiometric transition metal sulfides exhibit multifunctional characteristics. Owing to their exceptional electron transport properties and tunable surface-active sites, these materials simultaneously achieve the trifecta of high-precision sensing, efficient catalysis, and high-capacity energy storage. This unique combination opens new avenues for developing integrated or multifunctional energy devices. Future investigations should focus on elucidating the fundamental mechanisms governing this multifunctionality, thereby enabling the rational design of materials that seamlessly combine rapid charge-transfer kinetics with robust ion-intercalation capabilities. From an application perspective, scaling synthesis methods while maintaining precise structural control remains a critical hurdle. Additionally, integrating these materials into practical device architectures—such as flexible electrodes, hybrid electrolyzers, or tandem energy storage–conversion units—requires multidisciplinary approaches bridging materials science, engineering, and system design.

In summary, this work systematically reviews the synthesis strategies, structural diversity, and functional applications of NSTMS, highlighting their unique advantages over stoichiometric counterparts in terms of sensing, catalysis, and energy storage. The M_2_S_3_, M_6_S_8_, and M_6_S_6_ series deliver superior performance in flexible sensors, lithium-ion batteries, and electrocatalysis through defect engineering, intercalation, or doping of custom electronic structures. Advancements in synthesis techniques, such as liquid–liquid interface self-assembly, address key challenges in material preparation while opening up new opportunities for structural optimization. The article highlights the importance of several advanced characterization techniques, such as X-ray diffraction, scanning electron microscopy, and transmission electron microscopy, which play a crucial role in uncovering the crystal structure, surface morphology, and defect distribution of these materials. Furthermore, density functional theory calculations have become indispensable tools for predicting the electronic properties and catalytic behavior of these sulfides, providing valuable theoretical support and guidance. With these calculations, material design and optimization can be more precisely targeted to meet specific application needs. To harness the full potential of these materials, the article further explores their applications in sensors, ion batteries, and other energy storage devices, highlighting their advantages in improving device efficiency, extending lifespan, and enhancing stability. This review highlights the critical role of NSTMS in driving innovation in next-generation devices. Future research should focus on scalable synthesis, mechanistic studies of structure-property relationships, and integration into actual systems to maximize their potential. This work provides a basic perspective for the further exploration of NSTMS in functional materials science.
